# Nanostructures on Fluoropolymer Nanotextile Prepared Using a High-Energy Excimer Laser

**DOI:** 10.3390/ma16124280

**Published:** 2023-06-09

**Authors:** Petr Slepička, Nikola Slepičková Kasálková, Dominik Fajstavr, Bára Frýdlová, Petr Sajdl, Zdeňka Kolská, Václav Švorčík

**Affiliations:** 1 Department of Solid State Engineering, The University of Chemistry and Technology Prague, Technická 3, 166 28 Prague, Czech Republic; nikola.kasalkova@vscht.cz (N.S.K.); dominik.fajstavr@vscht.cz (D.F.); bara.frydlova@vscht.cz (B.F.); vaclav.svorcik@vscht.cz (V.Š.); 2 Department of Power Engineering, The University of Chemistry and Technology Prague, 166 28 Prague, Czech Republic; petr.sajdl@vscht.cz; 3 Faculty of Science, J. E. Purkyně University in Ústí nad Labem, 400 96 Ústí nad Labem, Czech Republic; zdenka.kolska@ujep.cz

**Keywords:** nanostructure, polymer, PTFE, silver nanolayer, morphology, wettability, laser exposure, antibacterial properties

## Abstract

This study is focused on polytetrafluoroethylene (PTFE) porous nanotextile and its modification with thin, silver sputtered nanolayers, combined with a subsequent modification with an excimer laser. The KrF excimer laser was set to single-shot pulse mode. Subsequently, the physico chemical properties, morphology, surface chemistry, and wettability were determined. Minor effects of the excimer laser on the pristine PTFE substrate were described, but significant changes were observed after the application of the excimer laser to the polytetrafluoroethylene with sputtered silver, where the formation of a silver nanoparticles/PTFE/Ag composite was described, with a wettability similar to that of a superhydrophobic surface. Both scanning electron microscopy and atomic force microscopy revealed the formation of superposed globular structures on the polytetrafluoroethylene lamellar primary structure, which was also confirmed using energy dispersive spectroscopy. The combined changes in the surface morphology, chemistry, and thus wettability induced a significant change in the PTFE’s antibacterial properties. Samples coated with silver and further treated with the excimer laser 150 mJ/cm^2^ inhibited 100% of the bacterial strain *E. coli*. The motivation of this study was to find a material with flexible and elastic properties and a hydrophobic character, with antibacterial properties that could be enhanced with silver nanoparticles, but hydrophobic properties that would be maintained. These properties can be used in different types of applications, mainly in tissue engineering and the medicinal industry, where water-repellent materials may play important roles. This synergy was achieved via the technique we proposed, and even when the Ag nanostructures were prepared, the high hydrophobicity of the system Ag-polytetrafluorethylene was maintained.

## 1. Introduction

PTFE has applications in a wide variety of industries, including the automotive, chemical, electronics, aerospace, and medical industries [1]. PTFE is commonly used to make films and coatings. These films and thin layers made of PTFE are then mainly used in the packaging industry. In the packaging of chemical equipment, pharmaceuticals, and cosmetic products, a chemically inert material such as PTFE is of an extraordinary cost compared to other commodity packaging materials [1]. The production of synthetic fibers using electrostatic forces has been performed for over a hundred years. The first fibers prepared in this way were carbon fibers. Polymeric man-made fibers have also been studied, but are problematic in terms of their drying and backing [2]. The preparation of nanofibers can be realized in several ways. The first of these is the pulling of fibers from a drop of polymer using a micropipette, when the micropipette is immersed in the polymer drop and the fiber is then pulled out at a speed of approximately 10^−4^ ms^−1^. This process is repeated several times and the individual fibers formed are deposited on the substrate. The disadvantage of this method is the small amount of usable polymers, since fibers can only be obtained from viscoelastic materials that tolerate deformation well [3]. Centrifugal spinning can also be used to prepare nanofibers. The principle of this is that several nozzles are filled with a polymer solution, then these nozzles are inserted into the sides of the rotating device, which overcomes the surface tension of the polymer solution with its high angular velocity.

Electrostatic spinning is a unique method that uses electrostatic forces to produce fine fibers. The fibers produced by this method have a small pore size and large surface area. The significant static charges in electrostatically produced fibers, which could be effectively manipulated to form three-dimensional structures, are applied [4]. Most charge carriers in organic solvents and polymers have lower mobilities, so the charge is expected to move longer distances throughout the liquid only if it has enough time to do so. After the liquid is ejected from the cone, the beam is directed to the charged collector, which collects the oppositely charged fibers [5]. Using the electrospinning method, it is possible to produce a nanofibrous PTFE textile [6,7]. PTFE nanofibres and nanofoils can be further activated via additive nanolayer formation [8] or plasma exposure [9]. Recently, perfluorethylenepropylene (FEP), with very similar structure to PTFE, has also been successfully used for honeycomb pattern formation as a basic substrate [10,11,12,13,14,15,16]. 

Our motivation was to prepare a Ag-PTFE nanocomposite with antibacterial properties, while the hydrophobic properties of this material would be maintained or even enhanced. The silver coating on flexible polymeric substrates, as an outstanding coating for metallic implants, was fabricated using a facile layer via the layer-coating method from Zhao et al. [17], where PTFE nanoparticles were immobilized in a sol–gel matrix and dip-coated onto 316 L of stainless steel via a mussel-inspired approach, followed by AgNP deposition. The application of polymer/nanosilver composite coatings for antibacterial applications was reviewed by Li et al. [18] in 2013, where the antibacterial functions of polymers were described in detail, and the combination of flexible polymers with nanosilver composite coatings for these antibacterial applications were confirmed to have excellent antibacterial properties. The decoration of polymeric substrates with a silver nanolayer has been reported previously for many different polymeric substrates, such as cellulose [19], polyethersulfone [20], or poly(N,N′-dimethylaminoethyl methacrylate) [21]. Polyethyleneimine/polyethersulfone nanofibrous membranes decorated with Ag nanoparticles [22] have exhibited excellent antibacterial properties against G+ and G− bacteria. The antibacterial and wound-healing applications of an imidazole-based porous organic polymer with a silver nanoparticles composite was presented by Zhou et al. [23]. An usual approach is to prepare silver-decorated polymeric material, but polymeric encapsulated silver nanoparticles have also been reposted to have antibacterial properties, proposed by people such as Rajanandkumar [24]. The antimicrobial activity of polymer/silver nanoparticles scaffolds is also supportive for the regeneration and growth of bone cells and tissue [25], where hydroxyapatite, presented in combination with a Ag polymer, shows a good biocompatibility, osteoconduction, and non-toxicity.

Laser treatment has been used to induce silver nanoparticles [26], which subsequently nucleated on PVA/PVP, thus forming polymeric blends doped with silver nanoparticles. Recently, as the primary substrate for Ag nanostructures, PTFE has also been used. Gou et al. published a study aimed at exploring the effects of surface modification on the adhesion enhancement of electrolessly deposited Ag-PTFE antibacterial composite coatings and their adhesion to silicon rubber substrates [27]. Wang et al. [28] prepared multifunctional cotton non-woven fabrics coated with silver nanoparticles and polymers for antibacterial, superhydrophobic, and high-performance microwave shielding. Some tremendous effects of silver on different polymeric substrates have already been published, but the approach of the laser-induced formation of a Ag-PTFE nanocomposite has not been published so far. Wang et al. was able to maintain the hydrophobic properties of silver-coated PTFE excellently, which was also our goal. Since laser dewetting procedures are well known to form an array of silver nanoparticles, we tried to expose the PTFE Ag nanoparticles with an excimer laser. Because our aim was to construct a silver nanoparticles/polymer nanocomposite, in the next paragraph, we would like to present a summary of the techniques used for this silver preparation and their characterization. 

Due to their unique physical and chemical properties, silver nanoparticles and nanostructures (AgNPs) are being increasingly being in various fields, such as healthcare, food, consumer, and industry. These properties include their optical, electrical, thermal, and other properties. Silver nanoparticles have recently been of great interest also due to their great antimicrobial activity [29]. Monodisperse AgNP samples can be synthesized by reducing silver nitrate with ethylene glycol in the presence of polyvinylpyrrolidone (PVP), the so-called polyol process [30]. An injection of the precursor solution into the hot solution is an effective means of inducing rapid nucleation in a short period of time, ensuring the production of AgNPs with smaller dimensions and a narrower size distribution [31]. The chemical synthesis process of AgNPs in solution uses three main components: metal precursors, reducing agents, and stabilizing agents. The initial nucleation and subsequent growth of nuclei can be regulated by adjusting the reaction parameters, such as: the reaction temperature, pH, precursors, reducing agents, and stabilizing agents [32,33]. The physical method of preparing metal nanoparticles is, in general, via evaporation or condensation processes, which can be carried out using a tube furnace at atmospheric pressure, but other physical-based methods for the synthesis of AgNPs have been developed [34]. A very common method for obtaining AgNPs is metal sputtering on a solid or liquid substrate. The most commonly used liquid substrates are vegetable oils or ionic liquids (ILs) [35]. The average size of these obtained AgNP particles is 9.5 nm [36]. An interesting method for obtaining AgNPs is metal sputtering into a liquid medium [37]. Nanoparticles are obtained via the direct photoreduction of metal, or a reduction in metal ions using photochemically generated intermediates, such as excited molecules and radicals—this is called photosensitization in the synthesis of nanoparticles [38]. One method for AgNP synthesis is the UV irradiation of a AgNO_3_^+^ carboxymethylated chitosan (CMCTS) solution. CMCTS serves simultaneously as a reducing agent for the silver cation and a stabilizing agent for the AgNPs in this method. It has also been found that the range of synthesized AgNPs is 2–8 nm; moreover, these nanoparticles can be dispersed in a CMCTS solution for 6 months [39]. In the case of a biological synthesis, AgNPs are the reducing agent and the stabilizers are replaced by living organisms (bacteria, algae, or fungi…) [40]. This method is economical, simple, reproducible, and requires less energy compared to chemical synthesis methods [41,42]. 

The applications of silver nanoparticles and nanostructures have generated interest in their biological properties, especially their high antibacterial activity [43]. In the field of medicine, silver nanoparticles are most commonly used in wound care as a cover to prevent infection, in surgical sutures and nets as additives serving as antibacterial agents, or in the form of coated dental implants [44]. Nanoparticles are also used for targeted drug transport, especially in oncological diseases. This method of treatment makes it possible to administer lower doses of a drug and, at the same time, reduces the risk of side effects of these drugs, because they are transported to and released only at the desired place [45]. To our best knowledge, the application of an excimer laser to a Ag-PTFE nanotextile, with significant changes in its surface morphology, strong hydrophobicity, and formation of a PTFE-Ag composite structure with a significant antibacterial effect, has not been presented until now.

## 2. Materials and Methods

### 2.1. Materials and Modification

The polymer substrate PTFE, in the form of nanotextiles from Goodfellow Ltd., Huntingdon, UK, was used for the experiments. The general properties of PTFE were a density of 2.2 g cm^−3^; a thickness of 45 μm, and a pore size of 0.2 µm. A Ag target from Safina a.s. (Vestec, Czech Republic) was used for the cathode sputtering (99.999% purity).

The sputtering was performed on Quorum Q150R S apparatus (Pfeiffer Vacuum, Aßlar, Germany) and silver layers of various thicknesses were sputtered on the PTFE samples—exposure times of 50 s; 100 s; 200 s; 300 s; 400 s; and 500 s were applied. The sputtering was performed under RT, with an argon pressure of 10 Pa, a sputtering current of 60 mA, and a distance from the sample of 50 mm.

The sputtered samples were selectively subjected to heat treatment in a Binder ED56 dryer (Helago, Hradec Kralove, Crech Republic). The heat treatment took place at 100 °C; 150 °C; and 200 °C for 1 h. 

The sputtered samples were exposed to a KrF excimer laser with a wavelength of 248 nm (Coherent Leap 100, Santa Clara, CA, USA) and an incident angle of 0°. The laser fluence was measured with a FieldMaxII-TOP device and set at 150 mJ/cm^2^ and 200 mJ/cm^2^. The single-shot pulse mode was chosen for this work.

### 2.2. Analytical Methods 

#### 2.2.1. Gravimetry 

The changes in the weights of the samples after the sputtering with silver were determined gravimetrically. UMX2 microbalances from the brand Mettler Toledo Automated–S were used for the gravimetric analysis. Before the actual weighing procedure, the samples were depolarized by a gate with a high-frequency field, because the charge on the sample could negatively affect the accuracy and correctness of this weighing. A total of four samples were weighed before the sputtering and after the sputtering and the arithmetic mean of the weight changes was used from the measured values. The equations used for the calculation of the average thickness were based on a simple equation between the layer thickness, area, mass, and density.

#### 2.2.2. Goniometry 

The contact angles between the distilled water and surfaces of the samples were measured on an ADVEX INSTRUMENTS goniometer (Brno, Czech Republic). A sample under examination was placed on a glass slide on a metal pad in front of the camera. In total, 8 drops of distilled water, with a volume of 5 μL ± 0.2, were applied to the surface of the sample with an automatic pipette. Using SEE System (Surface Energy Evaluation) software 6.3, all the drops were analyzed using the three-point method, which formed a circumscribed circle around the drop. The set of the contact angle values was statistically processed. We would like to emphasize that we measured and reported the apparent contact angles [46,47], but for the sake of clarity, we will further use the expression “contact angles” in the text. A Cassie-Bexter wetting regime was observed.

#### 2.2.3. Laser Confocal Microscopy 

Laser Confocal Microscopy—LCM (Laser Confocal Microscopy)—was performed on an Olympus LEXT OLS 300 confocal scanning microscope (Olympus, Prague, Czech Republic). LCM is an optical imaging technique that is able to display a 3D surface, thanks to the point scanning of the laser beam. Among the advantages of confocal microscopy is the possibility to place samples directly on the microscope stage—no vacuum is needed. 

#### 2.2.4. Atomic Force Microscopy

The surface morphology of the samples was analyzed using a DimensionIcon atomic force microscope (Bruker Corp., Billerica, MA, USA) in ScanAsyst mode. For this mode, a silicon nitride tip was used, operating near the resonant frequency of 70 kHz, with a spring constant of 0.4 Nm^−1^. To determine the surface roughness, the average roughness R_a_ was used, which represented the arithmetic mean of the deviations from the average surface of the sample. Additionally, an RMS was introduced, which represented the root mean square roughness and S, which represented an effective surface area of the sample. The obtained data were evaluated in the NanoScope Analysis program (version 1.80). 

#### 2.2.5. X-ray Electron Spectroscopy

The chemical compositions of the surfaces of the sputtered and laser-deposited samples were determined using X-ray photoelectron spectroscopy. The samples were irradiated with monochromatic X-rays with an energy of 1486.7 eV using an Omicron Nanotechnology ESCAProbeP spectrometer (Omicron nanotechnology GmbH, Taunusstein, Germany). From the results evaluated in the form of photoelectron spectra, the concentrations of elements (at. %) on the surfaces of the samples were obtained. These measurements were obtained with two positions—at 14° and 90° (90° represented a detection of photoelectrons perpendicularly to the surface). 

#### 2.2.6. Scanning Electron Microscopy and Energy Dispersive Analysis

The surface morphology of the samples was also determined using scanning (scanning) electron microscopy, which was performed on a LYRA3 GMU microscope (Tescan, Brno, Czech Republic). The measurement took place at a voltage of 5 kV. The elemental concentration and distribution were determined via energy dispersive spectroscopy (EDS) using an X-MaxN analyzer and 20 mm^2^ SDD detector (Oxford Instruments, Abingdon, UK) at a voltage of 10 kV. 

### 2.3. Antibacterial Tests

The antibacterial activity of the PTFE + Ag (50 s; 100 s; 200 s; 300 s; 400 s; and 500 s) + 150 mJ/cm^2^ samples was determined via a drop test using the Gram-negative bacteria *E. coli*. From agar plates of *E. coli* bacterial strains, one colony was transferred to 20 mL of liquid Luria–Bertani (LB) medium. The inoculum thus prepared was subsequently cultured overnight at 37 °C in an orbital shaker. The following day, the bacteria were diluted in sterile PBS to a concentration of approximately 1 × 10^3^ bacteria per 1 mL. The tested samples were placed in triplet on 2 mL of a bacterial suspension with the modified side and statically incubated at laboratory temperature. After 2 and 24 h, the bacterial suspension was mixed and five 25 μL drops of each were pipetted onto LB agar culture dishes. These dishes were cultured overnight at room temperature. Subsequently, the number of colony-forming units (CTUs) was determined, which was compared with the number of CTUs in the control. The experiment was performed under sterile conditions. 

## 3. Results

### 3.1. Thickness of Sputtered Layers

**Figure 1:**
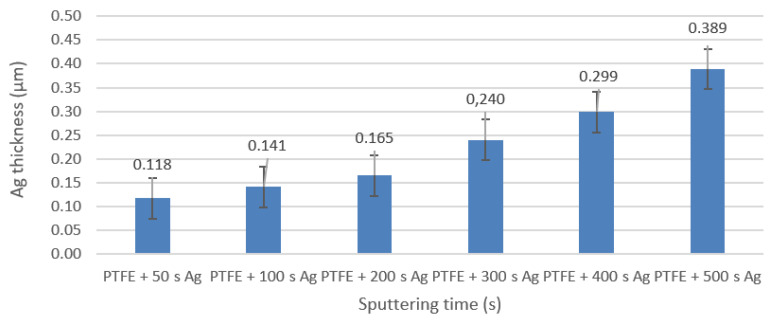
Dependence of the thickness of the Ag layer on the deposition time on PTFE determined gravimetrically.

Using gravimetry, the thicknesses of the silver layers were determined after different sputtering times of the PTFE substrate (50 s; 100 s; 200 s; 300 s; 400 s; and 500 s). As expected, the Ag layer thickness values increased with an increase in the sputtering time. This method was mainly used for a sample quantification of the silver layer thickness, with possible inaccuracies in the form of the PTFE material’s density (as it is a porous nanotextile, the density of the sample was likely to differ from the table value) and in the form of the powdered Ag’s density (whose density in the form of powdered thin layer slightly decreased) (Figure 1).

### 3.2. Wettability

The wettability of the surfaces of the pristine PTFE samples, PTFE samples with a silver layer, PTFE samples with sputtered silver and laser exposure at 150 mJ/cm^2^, and PTFE samples with a silver layer and laser exposure at 200 mJ/cm^2^ was studied goniometrically by measuring the contact angle of the distilled water. In the following graph, the influence of the type of sample modification on their wettability can be seen.

**Figure 2:**
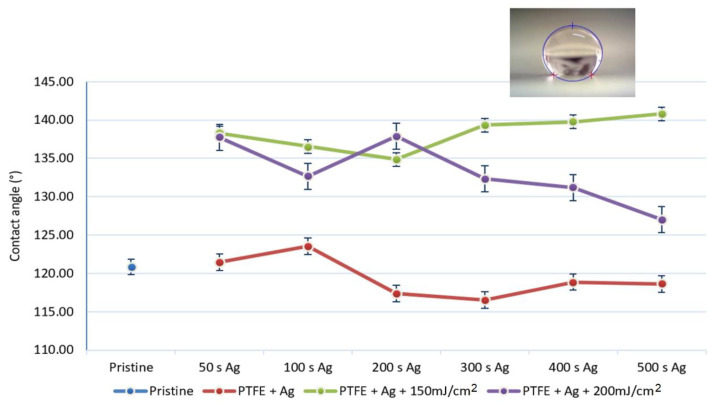
Results of the goniometric determination of the surface wettability of the samples, the samples were sputtered with Ag with sputtering time 50–500 s, and subsequently treated with an excimer laser and laser fluence 150 mJ/cm^2^ and 200 mJ/cm^2^.

It can be seen from Figure 2 that the laser-exposed samples were, in all cases, more hydrophobic than the pristine samples and Ag sputtered samples without laser modification. This behavior was probably caused by the creation of Ag nanoparticles and a Ag/PTFE composite of a specific spherical shape using an excimer laser, which were then physically incorporated into the structure of the porous PTFE substrate. The lowest average contact angle was for the PTFE + 300 s Ag (116.5°) sample and the highest average contact angle was measured for the PTFE + 500 s Ag + 150 mJ/cm^2^ (140.8°) sample. Such high values were close to being stated as superhydrophobic, which is a little surprising for a Ag-based surface, but this was probably caused by their unique morphology, which will be discussed later. The hydrophobicity, combined with the Ag surface, may also have impaired the ability of the cell and bacterial adhesion [28], so it could be assumed that the chosen combination—silver with hydrophobic surface properties—may have interesting antibacterial properties. On the basis of our experiment (nanotextiles were suspended in a bacterial solution and the bacteria amount was counted after 24 h), this could only be assumed. The following figure shows a drop of distilled water on a substrate of PTFE + 500 s Ag + 150 mJ/cm^2^.

### 3.3. Laser Confocal Microscopy

**Figure 3:**
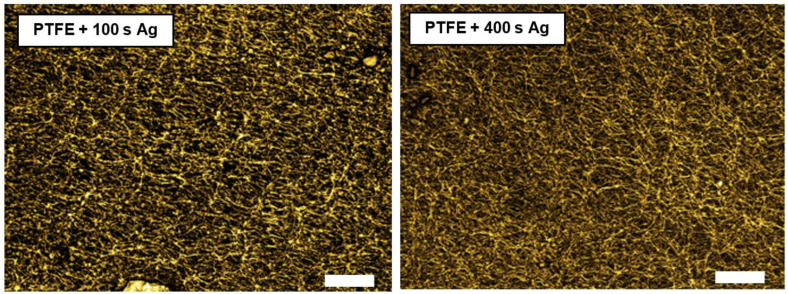
Images from laser confocal microscopy (LCM) of PTFE sputtered Ag samples 100 s (**left**) and 400 s (**right**) (scale bar 15 μm).

The surface morphology of the PTFE samples was also studied with a confocal scanning microscope (Figure 3). The measurements included the PTFE + Ag samples; PTFE + Ag + 100 °C; PTFE + Ag + 200 °C; PTFE + Ag + 150 mJ/cm^2^; and PTFE + Ag + 200 mJ/cm^2^. The sample images were taken in CF mode. The first set of studied samples was chosen to be PTFE coated with Ag. The following selected images show the PTFE + 100 s Ag and PTFE + 400 s Ag samples at a 1200× magnification. The images show the morphology of the PTFE porous nanotextile upon which the silver was sputtered. However, the silver sputtering time had only a slight effect on the surface morphology of the samples, which was also related to the slight change in the goniometry described in previous section.

**Figure 4:**
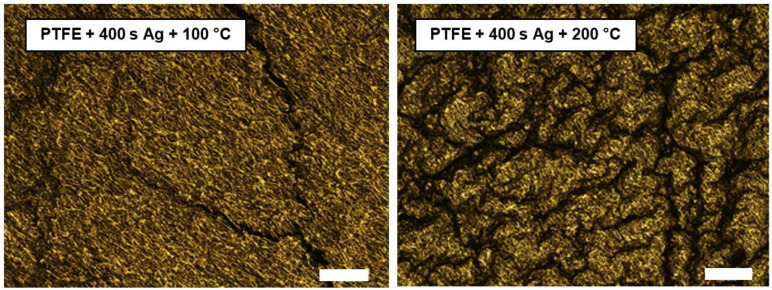
Images from laser confocal microscopy (LCM) of PTFE sputtered and heat-treated samples—Ag 400 s, 100 °C (**left**); Ag 400 s, 200 °C (**right**) (scale bar 15 μm).

The images in Figure 4 show the PTFE samples sputtered for 400 s with heat treatments of 100 °C and 200 °C at a 1200× magnification. The images show very similar morphologies of the PTFE nanofibers to those in Figure 3. However, the fundamental difference is the visible depressions; thus, there was a change in the morphology, especially on the sample exposed to 200 °C thermal stress. Even though PTFE itself is stable at a temperature of 200 °C, a combination with sputtered silver caused the samples to become scratched, due to different bilayer (PTFE/Ag) heat conductivity along their edges. 

**Figure 5:**
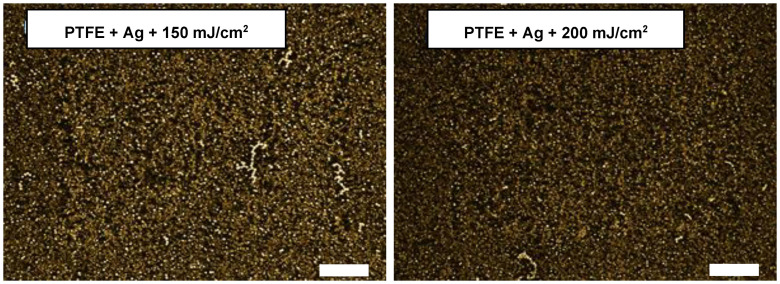
Images from laser confocal microscopy (LCM) of PTFE sputtered and laser-exposed samples–Ag 400 s, 150 mJ/cm^2^ (**left**); Ag 400 s, 200 mJ/cm^2^ (**right**) (scale bar 15 μm).

The final set of studied samples exhibited the influence of the high-energy excimer laser treatment. The chosen samples were sputtered for 400 s with Ag and subsequently treated with an excimer laser—therefore, the surface morphology of the PTFE + Ag + 150 mJ/cm^2^ and PTFE + Ag + 200 mJ/cm^2^ samples are introduced in Figure 5.

The excimer-laser-exposed images show a significantly different morphology from the sputtered samples and sputtered and thermally stressed samples. Instead of the morphology of the porous PTFE with sputtered silver, as was the case in Figure 4, it is noticeable that the morphology changed (see Figure 5). Protruding clusters and nanostructures of silver and Ag/PTFE, which were not present without this laser modification, can be seen. Silver nanoparticles were created with an excimer laser and incorporated into a nanofibrous PTFE nanotextile, which expanded. These changes will be shown in more detail in the next section. These significant changes were also reflected in the changes in wettability, as seen in the previous section “Wettability”, where a significant increase in the contact angles could be observed for the excimer-exposed samples.

### 3.4. Atomic Force Microscopy

**Figure 6:**
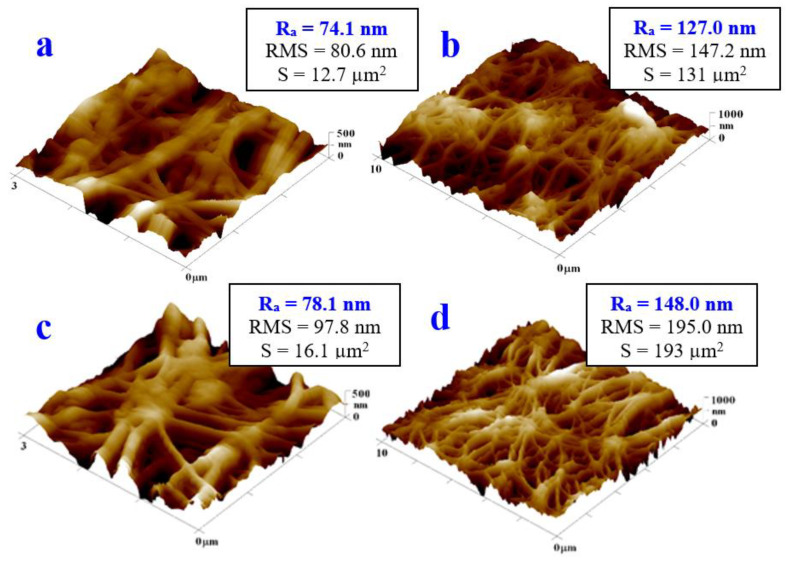
Surface morphology of pristine PTFE samples (square 3 × 3 μm^2^ (**a**) and 10 × 10 μm^2^ (**b**)) and samples exposed with excimer laser with fluence 150 mJ/cm^2^ (square 3 × 3 μm^2^ (**c**) and 10 × 10 μm^2^ (**d**)) determined with atomic force microscopy.

The surface modifications of the pristine PTFE, those with sputtered silver, and those subsequently exposed to the excimer laser were also studied using atomic force microscopy (AFM). The first set of samples was focused on the surface of the pristine PTFE and laser-treated PTFE samples without the silver sputtering. From the AFM images of the pristine PTFE (Figure 6, first line), its structure is evident, which can be described as porous nanotextile material. The value of R_a_ slightly differed as a result of the non-uniform arrangement of the fibers in the structures of the samples. The detected morphology was in good agreement with that observed using the laser confocal microscopy. As another set of studied samples, we chose the PTFE treated with the excimer laser only (PTFE + 150 mJ/cm^2^), so we could compare the influence of the excimer on the PTFE itself and compare it with those covered with Ag (Figure 6, second line). 

From the AFM images of the PTFE + 150 mJ/cm^2^ sample, a significant similarity to the pristine PTFE samples was observed. The value of Ra increased slightly, but, as already mentioned, the reason for this was the non-uniform distribution of the fibers and pores of the PTFE material. An interesting result was that, despite the relatively high energy of the excimer laser exposure, the PTFE material remained morphologically (and chemically) unchanged, which was subsequently confirmed even for the same energy and energy of up to 6000 pulses, so it was insensitive to the excimer beam and could be used as an optimal substrate for modifications of additively deposited layers. 

**Figure 7:**
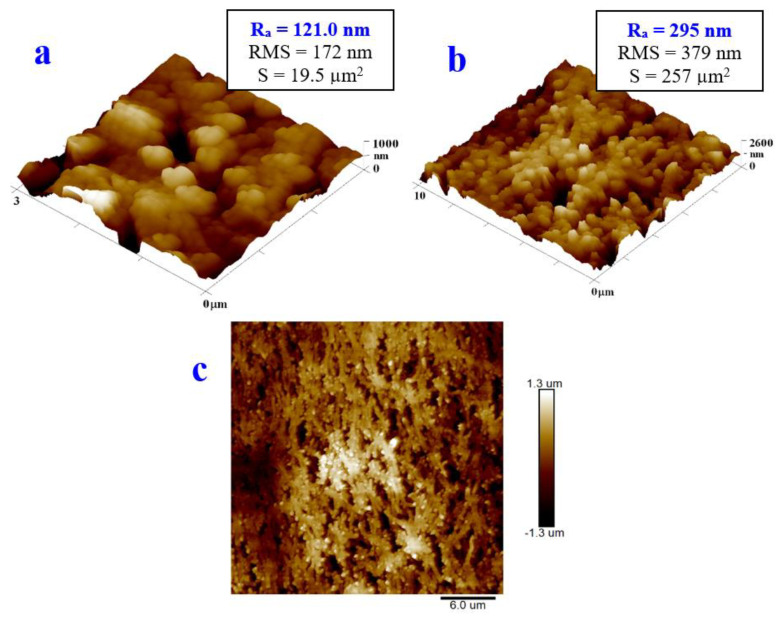
Surface morphology of silver-coated (Ag 300 s) PTFE samples exposed with excimer laser exposure with fluence 150 mJ/cm^2^ (square 3 × 3 μm^2^ (**a**) and 10 × 10 μm^2^ (**b**)) determined with atomic force microscopy. 2D image of the square 30 × 30 μm^2^ is also provided (**c**).

The morphological and chemical changes in the PTFE material became apparent only after its deposition with silver and subsequent exposure with an excimer laser. The combination of silver and the excimer laser treatment on the PTFE substrate induced interesting composite structures, with silver nanoparticles incorporated (physically) into the porous structure of the PTFE nanotextile (Figure 7). This process thereby induced on this material properties such as a high hydrophobicity, which was described in the previous section and could also be used for antibacterial surfaces, which will be described further in this paper. 

**Figure 8:**
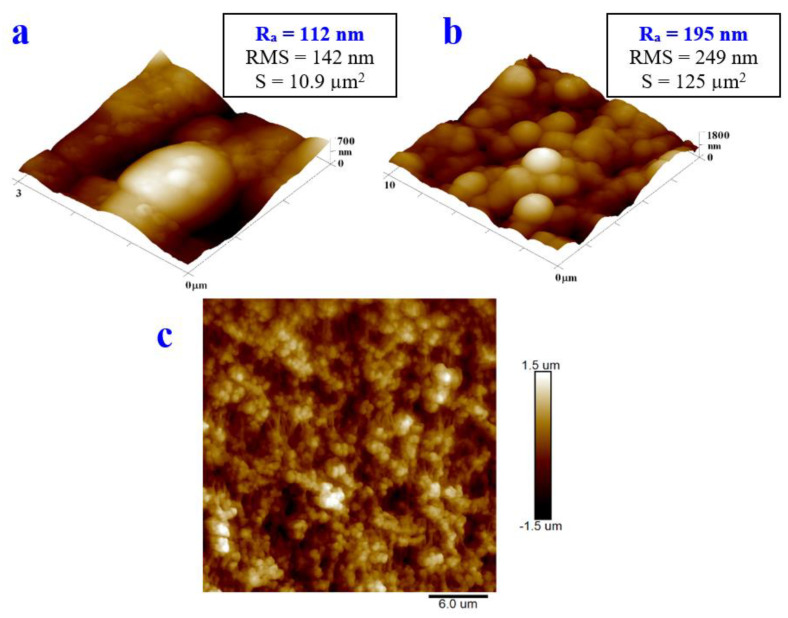
Surface morphology of silver-coated (Ag 500 s) PTFE samples exposed with excimer laser exposure with fluence 150 mJ/cm^2^ (squares 3 × 3 μm^2^ (**a**) and 10 × 10 μm^2^ (**b**)) determined with atomic force microscopy. 2D image of the square 30 × 30 μm^2^ is also provided (**c**).

The samples of the PTFE + 300 with Ag showed a significantly higher value of R_a_ than that of the samples of the pristine PTFE. The surface morphology changed in such a way that additional globular-shaped layers could be observed on the PTFE nanotextile substrate, which were formed by layers of powdered silver and the composite with PTFE (Figure 7 and Figure 8). On the 2D image, the nanofibrous structure of the PTFE substrate, with protruding globular particles of silver and the Ag/PTFE composite, are excellently visible.

The last sample to compare is the PTFE sputtered with silver (500 s) that was subsequently treated with the excimer laser—the “PTFE + Ag 500 s + 150 mJ/cm^2^” sample. From Figure 7, it is evident that the excimer laser induced the creation of Ag nanoparticles and their incorporation into the PTFE structure. Here, (Figure 8), we can see the same change in morphology, but the globular structure is more pronounced, with a larger dimension of Ag nanoclusters.

### 3.5. Scanning Electron Microscopy

**Figure 9:**
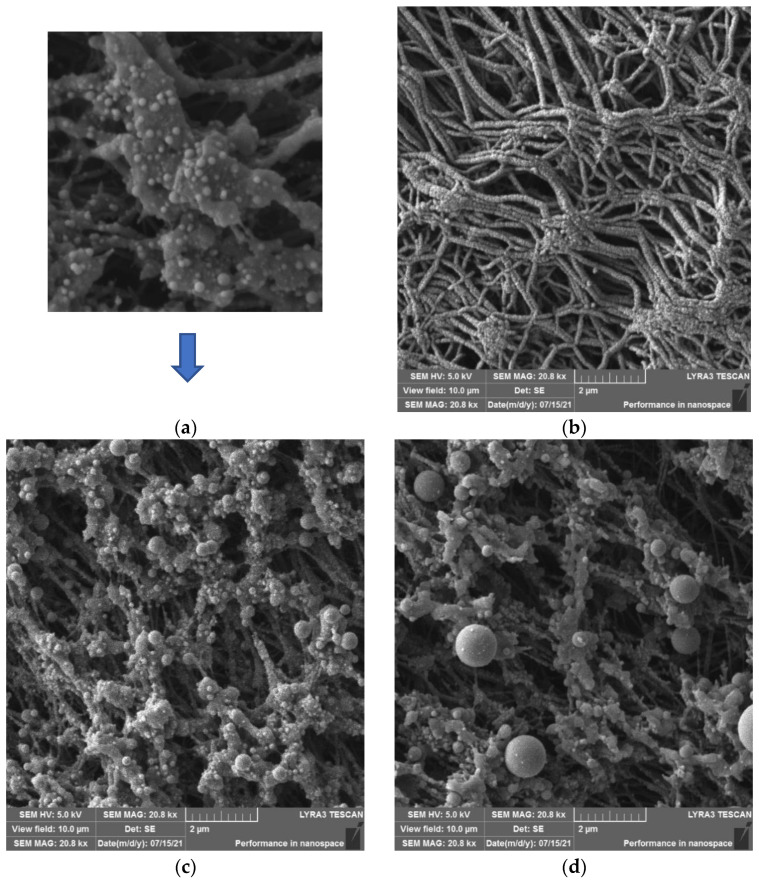
Detailed SEM image of PTFE nanotextile with sputtered Ag 300 s and subsequently treated with 150 mJ/cm^2^ with visible Ag nanoparticles (**a**), SEM images of PTFE nanotextile sputtered with Ag 400 s (**b**), PTFE nanotextile with sputtered Ag 300 s and subsequently treated with 150 mJ/cm^2^ (**c**), and PTFE nanotextile with sputtered Ag 400 s and subsequently treated with 150 mJ/cm^2^ (**d**).

Scanning electron microscopy (SEM) was used to obtain an even more concrete idea of the surface morphology of the modified PTFE substrates. The set of samples studied included (both SEM and EDS analyses) the PTFE + Ag (50–500 s) and PTFE + Ag (50–500 s) + 150 mJ/cm^2^ samples. We have selected for the SEM presentation the PTFE + Ag 400 s and 500 s samples (see Figure 9).

### 3.6. Surface Chemistry–XPS and EDS Analysis

The elemental representations on the surfaces of the studied samples were determined using X-ray photoelectron spectroscopy (XPS). The samples studied using this method were the PTFE + Ag (50 s; 100 s; 200 s; and 500 s) + 150 mJ/cm^2^ and PTFE + Ag (50 s; 100 s; 200 s; and 500 s) + 200 mJ/cm^2^ samples. The following table will summarize the results of this XPS analysis of the samples (see Table 1).

The table shows a clear trend of increasing at.% silver on the surface of the PTFE samples with an increasing sputtering time. This trend is obvious and thus confirms the quantitative analysis from Section 3.1. Oxygen, which is represented in the range of 1.0–5.2%, was detected due to the oxidation of the silver layer (see Table 2).

The chemical composition and distribution of elements on the surfaces of the PTFE + Ag (50 s; 300 s; and 500 s) and PTFE + Ag (50 s; 300 s; and 500 s) + 150 mJ/cm^2^ samples were studied using energy dispersive spectroscopy (EDS). Therefore, there were certain differences between the at. % measured using both methods. The reason for this was that there was a limited amount of silver sputtered onto the PTFE nanotextile, and due to the excimer laser exposure, the nanocoposite Ag-PTFE was formed. The amount of silver was distributed into the expanded PTFE matrix. As the XPS method revealed information from several atomic layers (ca 1 nm), and EDS could acquire information from higher depth (up to hundreds of nm), the EDS concentrations were detected higher compared to those acquired via the XPS technique. It is evident that the laser exposure induced the Ag/PTFE composite, which is supported by the decrease in the Ag after the application of the laser for the substrates sputtered with 300 and 500 s, as the Ag was “mixed” with the PTFE bulk material.

### 3.7. Antibacterial Tests

The study of the surfaces’ physico-chemical changes on the treated PTFE was conducted with the main aim of inducing the antibacterial properties of such polymeric materials. 

**Figure 10:**
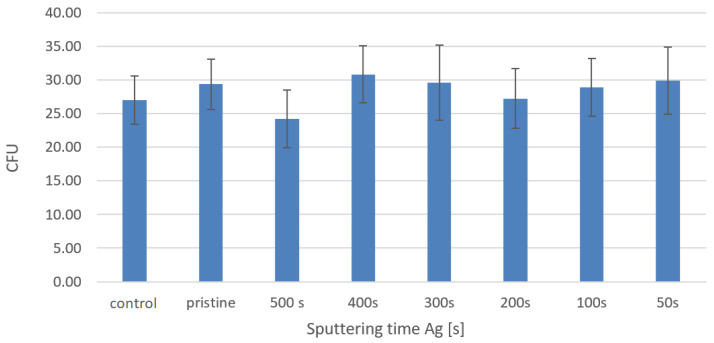
Number of CFUs after two hours of leaching for *E. coli* bacterial suspension on PTFE + Ag samples (50–500 s) + 150 mJ/cm^2^.

**Figure 11:**
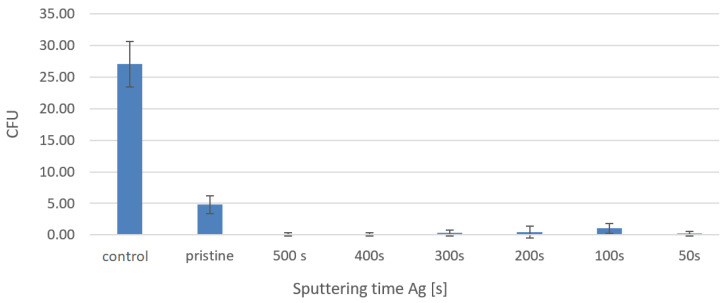
Number of CFUs after 24 h of leaching for *E. coli* bacterial suspension on PTFE + Ag samples (50–500 s) + 150 mJ/cm^2^.

We conducted our antibacterial experiments with the strain *Escherichia coli* (*E. coli*). The entire procedure for carrying out these antibacterial tests was described in the Experimental section. The first determination of the number of colony-forming units (CFU) was performed after two hours of leaching the bacterial suspension on the studied samples (Figure 10). It can be seen from graph that, after two hours of soaking the samples in the bacterial suspension, the samples did not show any significant antibacterial properties. A dramatically different situation occurred after the 24 h from the leaching, which is well documented in Figure 11.

It can be summarized from Figure 11 that, after 24 h of soaking the samples in a bacterial suspension, their antibacterial properties decreased very significantly, both for the sputtered samples and for the pristine PTFE sample. In the case of the PTFE + 500 with Ag + 150 mJ/cm^2^ sample, the number of CFUs even dropped to zero. As discussed earlier, the surface morphologies related to goniometry may also have been related to the antibacterial properties of the material. We have to honestly admit that, in our case, it can be only assumed that a high hydrophobicity contributed to the antibiofouling surface and thus decreased the ability of the bacteria adhesion to the material surface, on the basis of the previously acquired data from Wang et al. [28].

## 4. Conclusions

This work was focused on the study of PTFE porous nanotextile and the preparation of thin silver layers, with a subsequent modification using an excimer laser. Minor effects of the excimer laser on the pristine PTFE substrate were described, but very significant effects of the excimer laser on the PTFE + sputtered silver system were achieved. The excimer treatment induced the formation of a silver nanoparticles/PTFE/Ag composite with a wettability close to super hydrophobicity. The EDS and XPS methods showed the elemental changes on the surface of the modified PTFE samples. These changes confirmed the aforementioned morphological changes. Both scanning electron microscopy and atomic force microscopy revealed the formation of superposed globular structures on the PTFE lamellar primary structure, which was also conformed using energy dispersive spectroscopy. Combined changes in the surface morphology, chemistry, and thus wettability induced a significant change in the PTFE’s antibacterial properties. Antibacterial tests showed that the sputtered and laser-modified samples had excellent antibacterial properties. The samples coated with silver and further treated with the excimer laser at 150 mJ/cm^2^ inhibited 100% of the bacterial strain *E. coli*.

## Figures and Tables

**Table 1:** Atomic concentration of the sample surface obtained using the XPS method, the analysis was performed by detecting the outgoing beam at angles of 14° and 90° (perpendicular to the sample surface).

Sample	Concentration (at.%)			
	C (1 s)	O (1 s)	F (1 s)	Ag (3 d)
Angle 14°				
PTFE/Ag50 s/150 mJ/cm^2^	38.2	5.2	52.4	4.2
PTFE/Ag100 s/150 mJ/cm^2^	30.5	1.4	61.5	6.7
PTFE/Ag200 s/150 mJ/cm^2^	36.6	1.5	54.4	7.5
PTFE/Ag500 s/150 mJ/cm^2^	41.4	5.2	42.6	10.8
PTFE/Ag50 s/200 mJ/cm^2^	36.8	1.3	57.7	4.3
PTFE/Ag100 s/200 mJ/cm^2^	36.4	3.4	54.9	5.2
PTFE/Ag200 s/200 mJ/cm^2^	42.5	1.0	51.1	5.4
PTFE/Ag500 s/200 mJ/cm^2^	36.0	2.5	50.6	10.9
Angle 90°				
PTFE/Ag50 s/150 mJ/cm^2^	33.5	1.1	59.5	5.9
PTFE/Ag100 s/150 mJ/cm^2^	32.8	3.6	56.1	7.5
PTFE/Ag200 s/150 mJ/cm^2^	29.7	4.6	58.0	7.8
PTFE/Ag500 s/150 mJ/cm^2^	30.7	5.0	54.1	10.2
PTFE/Ag50 s/200 mJ/cm^2^	34.9	3.5	56.9	4.7
PTFE/Ag100 s/200 mJ/cm^2^	34.5	2.1	58.2	5.2
PTFE/Ag200 s/200 mJ/cm^2^	35.3	3.9	55.2	5.6
PTFE/Ag500 s/200 mJ/cm^2^	36.5	4.2	49.3	10.0

**Table 2:** Atomic concentration of the surface of the samples obtained by the EDS method.

Sample	Concentration (at.%)			
	C	O	F	Ag
PTFE/Ag50 s	40.5	0.5	47.9	11.1
PTFE/Ag300 s	34.1	0.9	29.8	35.2
PTFE/Ag500 s	26.2	6.4	24.8	42.7
PTFE/Ag50/150 mJ/cm^2^	34.8	0.5	50.8	13.9
PTFE/Ag300 s/150 mJ/cm^2^	46.5	0.2	46.1	7.1
PTFE/Ag500 s/150 mJ/cm^2^	40.9	0.6	37.8	20.6

## Data Availability

The data presented in this study are available on request from the corresponding author.
